# A Rapid and Reliable Test for *BRCA1* Promoter Hypermethylation in Paraffin Tissue Using Pyrosequencing

**DOI:** 10.3390/diagnostics15050601

**Published:** 2025-03-01

**Authors:** Ruben Bacares, Robert Soslow, Narciso Olvera, Douglas A. Levine, Liying Zhang

**Affiliations:** 1 Departments of Pathology, Memorial Sloan Kettering Cancer Center, New York, NY 10065, USA; bacaresr@mskcc.org (R.B.); soslowr@ccf.org (R.S.); 2 Department of Pathology, Cleveland Clinic, 9500 Euclid Avenue, Cleveland, OH 44195, USA; 3 Departments of Surgery, Memorial Sloan Kettering Cancer Center, New York, NY 10065, USA; narciso.olvera@nyulangone.org (N.O.);; 4 Laura and Issac Perlmutter Cancer Center, NYU Grossman School of Medicine, NYU Langone Health, New York, NY 10016, USA; 5 Global Clinical Development, Merck Research Laboratories, Rahway, NJ 07065, USA; 6 Department of Pathology and Laboratory Medicine, David Geffen School of Medicine, University of California, Los Angeles, CA 90095, USA

**Keywords:** BRCA, hypermethylation, pyrosequencing

## Abstract

**Background:** Ovarian cancers harboring inactivating mutations in *BRCA1* or *BRCA2* demonstrate increased sensitivity to poly (ADP-ribose) polymerase inhibitors (PARPis). *BRCA1* promoter methylation could serve as a more precise biomarker for therapy response, as it reflects a dynamic mechanism, compared with genomic scarring, which remains persistent and lacks real-time prediction of sensitivity after prior lines of treatment. Additionally, the *BRCA1* promoter methylation may provide a more precise biomarker for identifying homologous recombination deficiency compared to genomic scars. In this study, we describe the validation of a pyrosequencing method to assess *BRCA1* promoter methylation status. **Methods:** Tumor DNA from high-grade serous ovarian carcinoma was tested targeting 11 CpG sites adjacent to the *BRCA1* transcription start site. All cases had concordant results compared with TCGA methylation data or real-time PCR results. To determine the sensitivity of this assay, we performed a dilution series experiment using seven mixtures of methylated DNA and unmethylated genomic DNA (100%, 50%, 25%, 12.5%, 6.25%, 3.125%, and 1.56%). **Results:** We observed a high degree of correlation (R^2^ = 0.9945) between predicted and observed results. Intra- and inter-run reproducibility was established by performing six cases in triplicate in the same run and in three different runs. **Conclusions:** By applying 10% as the cutoff for detection of methylation, the PyroMark Q24 pyrosequencing assay demonstrated 100% concordance across all the ovarian cancer cases included in this validation. This assay has been approved by the New York State Department of Health as a laboratory-specific assay for clinical use.

## 1. Introduction

The American Cancer Society estimates that approximately 19,680 women were diagnosed with ovarian carcinoma (OC) in 2024 and approximately 12,740 patients died from this disease [1]. High-grade serous carcinoma (HGSC) is the most prevalent histologic sub-type of OC, accounting for approximately two-thirds of OC deaths. Although recent advances in systemic therapies have extended survival, there is still a need to further refine treatments for advanced ovarian carcinoma to enhance long-term survival and cure rates. In recent years, poly (ADP-ribose) polymerase inhibitors (PARPis) have become part of the standard of care for the treatment of ovarian cancer. Cells with inactivating mutations of the *BRCA1* or *BRCA2* tumor suppressor genes show increased sensitivity to PARPis. The first data demonstrating a benefit in overall survival (OS) from maintenance therapy with PARPis were reported following a seven-year follow-up period. This is a significant discovery for the 20–22% of ovarian cancer patients who carry a somatic or germline BRCA mutation (g/sBRCAm) [2].

The promoter hypermethylation of the *BRCA1* gene results in a loss of expression of BRCA1 in ovarian cancer. *BRCA1*-methylated OC displays similar clinicopathological features to *BRCA1*-mutated OC, which suggests molecular similarities between mutation- and epigenetic-induced *BRCA1* silencing. Patients with ovarian cancer with high levels of *BRCA1* hypermethylation are very likely to have high genomic instability scores, which may sensitize tumors to PARPi treatment [3,4,5]. Thus, the determination of the methylation status of the *BRCA1* promoter is pressingly needed for the prediction of responses to PARPis in order to select patients for Food and Drug Administration (FDA)-approved therapies and stratify patients into clinical trials.

A variety of techniques for the study of DNA methylation, such as methylation-specific PCR (MSP) and MethyLight real-time PCR, have been developed for *BRCA1* methylation analysis. In MSP, specific primers are designed to amplify regions of interest. A key feature of this method is the use of separate primers that selectively amplify either methylated or unmethylated alleles of the target sequence. Therefore, the traditional MSP is primarily a qualitative technique, providing information on the presence or absence of DNA methylation at specific genomic sites. In contrast, quantitative MSP, when coupled with real-time PCR, offers semi-quantitative data, enabling the assessment of relative methylation levels. This approach was recently employed in a study examining *BRCA1* promoter methylation in breast cancer [6]. MethyLight real-time PCR offers several advantages, such as high sensitivity and quantification of methylation; however, it does have some notable disadvantages such as technical complexity, including the need for careful primer design and PCR optimization and limited ability to analyze broad methylation patterns across the genome. The Illumina methylation array is a high-throughput platform used to measure DNA methylation across the genome. It uses a BeadChip array to analyze methylation at specific CpG sites, providing an efficient and comprehensive method for studying epigenetic modifications. However, the method comes with disadvantages, including its complexity and high cost, as it requires a separate scanner for data acquisition [7]. Pyrosequencing is another method that has received attention for the simultaneous analysis and quantification of the degree of methylation at multiple CpG positions in proximity. Pyrosequencing is ideally suited for DNA methylation analysis after bisulfite treatment of DNA because it offers several advantages compared to other methods: (1) it allows the interrogation of multiple consecutive CpG sites concurrently; (2) it provides for real-time monitoring of the sequencing process, providing immediate results without the need for extensive downstream analysis; (3) it involves fewer steps than other methods, so results can be generated 15 min after the plate is placed in the pyrosequencer; (4) this assay is inexpensive compared with a DNA methylation array. Because of these features, pyrosequencing has been widely used in clinical laboratories for mutation detection [8]. In this study, we present the validation of a pyrosequencing method for assessing *BRCA1* promoter methylation.

## 2. Materials and Methods

We identified twenty (20) HGSC cases that were previously analyzed as part of The Cancer Genome Atlas (TCGA) project [9] for methylation events using the Illumina methylation array (Illumina, San Diego, CA, USA), or for *BRCA1* methylation analysis by a real-time PCR assay developed by our research team at Memorial Sloan Kettering Cancer Center (MSKCC) as part of other ongoing laboratory studies. Three cases had overlapping data generated by both methods. The 20 cases were intentionally selected for assay development with an even distribution; 10 cases were previously found to be *BRCA1*-methylated and 10 cases were found to be unmethylated. Tumor DNA was treated with proteinase K during extraction to completely remove residual amounts of proteins that may inhibit complete bisulfite conversion. The quality and quantity of genomic DNA preparation is essential for successful bisulfite conversion. A 260/280 ratio of 1.6 to 2.5 was set for DNA quality and all samples met these criteria.

Bisulfite treatment of DNA was performed using the Zymo EZ-DNA Methylation-Direct™ Kit following manufacturer’s instructions (Zymo Research, Irvine, CA, USA, D5020). A total of 500 ng of DNA was used for each treatment. A positive control specimen (CpGenome universal methylated DNA, Cat# S7821, Millipore Corporate, Billerica, MA, USA) and a negative control specimen (peripheral blood DNA) were used for QC in each run to ensure complete bisulfite conversion. We used universally methylated DNA initially, but we also used patient samples, as they more accurately reflect the clinical lab setting [i.e., they often have poor quality templates due to extraction from formalin-fixed and paraffin-embedded (FFPE) tissues]. After bisulfite treatment, unmethylated Cs are converted to Ts.

After bisulfite treatment, all samples were PCR-amplified using primers for the *BRCA1* promoter region. The sequence of the forward primer was 5′-GTATTTTGAGAGGTTGTTGTT-3′. The sequence of the reverse primer was 5′-ATCTAAAAAACCCCACAACCTA-3′. Each PCR reaction contained 5 μL 10× Qiagen Buffer with 15 mM MgCl_2_ (Qiagen, Valencia, CA, USA)), 0.3 μL 5 U/μL ABI Taq Gold (Applied Biosystems, Carlsbad, CA, USA, part number: N8080245), 4 μL 10 mM dNTPs (Invitrogen Corporation, Carlsbad, CA, USA, catalog number: 18427-088), 2 μL 100 ng/μL primers (1 μL for each), 100 ng of bisulfite-treated DNA, and water to make the final volume of 50 μL. Cycling conditions were 95 °C for 15 min, 95 °C for 20 s (45×), 60 °C for 20 s (45×), and 72 °C for 20 s (45×), with a final extension at 72 °C for 5 min (1×). The anticipated product size was 151 bps.

**Figure 1:**
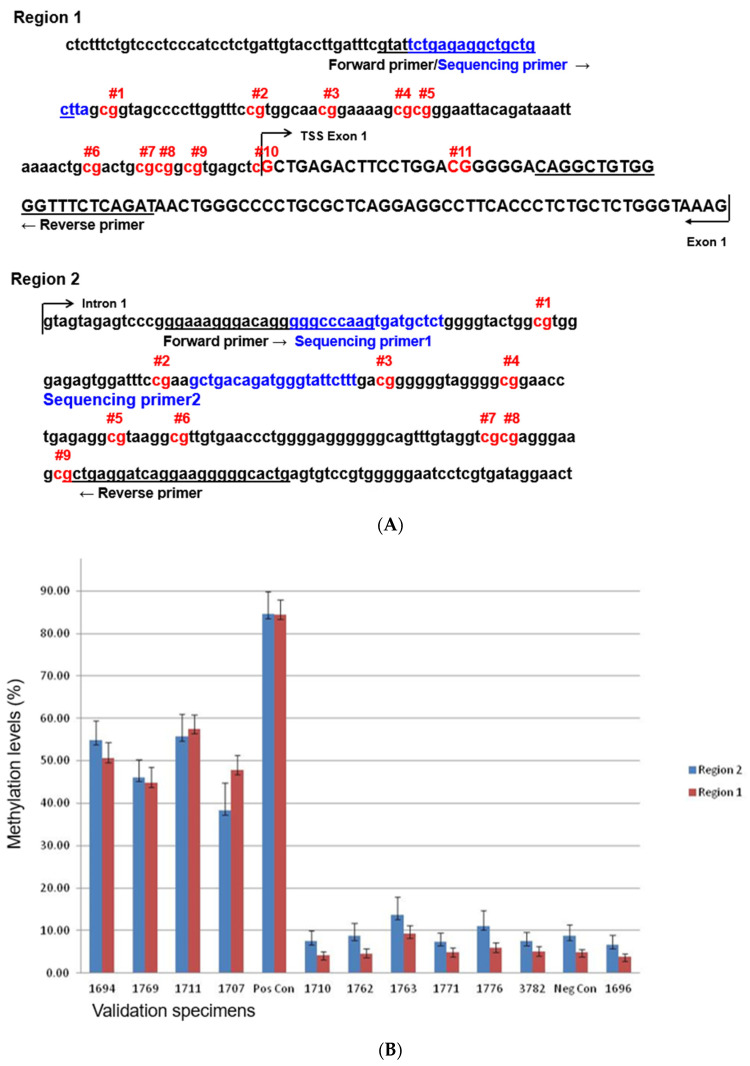
Comparison of the *BRCA1* methylation levels of two promoter regions. The uppercase letters correspond to the 5′ untranslated region (5′UTR) between the transcription start site (TSS) and the start codon in the mRNA sequence. In the case of *BRCA1* (RefSeq ID: NM_007294.4), exon 1 does not encode proteins, as the ATG is in exon 2 of *BRCA1*. The lowercase letters are promoter sequences that are not transcribed. (**A**): Two promoter regions of *BRCA1*. The forward and reverse primers used for PCR are underlined. The sequencing primer is in blue (note, the forward PCR primer and sequencing primer overlap with each other). The CpG sites interrogated by this assay are shown in red. Region 1 was previously published [10] and region 2 is based on our internal data. Region 2 is immediately downstream of region 1. (**B**): Comparison of these two regions using a set of samples that were previously tested for *BRCA1* promoter methylation. TSS: transcription start site.

The PCR products were subjected to pyrosequencing on a PyroMark Q24 Pyrosequencer (Qiagen) using in-house-developed primers targeting 11 CpG sites adjacent to the *BRCA1* transcription start site (Figure 1, Region 1). The sequence of the sequencing primer is 5′-TTTGAGAGGTTGTTGTTTA-3′. Sequence to be analyzed is 5′-gc/tggtagttttttggttttc/tgtggtaac/tggaaaagc/tgc/tgggaattatagataaattAAAATTG C/TGATTGC/TGC/TGGC/TGTGAGTTC/TGTTGAGATTTTTTGGAC/TGGGGGATAGGTTGTGGGGTTTTTTAG and nucleotide dispensation order is TGTCGTAGTTAGTTCGTGCTGATCGATGTCGTCGATATAGATATAATAGTCGATGTCGTCGT CGTGAGTCGTGAGATTGATCGGAT.

The pyrosequencing assay was designed to interrogate the methylation status of 11 CpG sites in region 1. The degree of methylation of each CpG site was calculated using the following formula: methylation % = [peak height methylated/(peak height methylated + peak height non-methylated)] × 100. This is achieved using the allele quantification functionality of the PyroMark Q24 software and can be exported for further analysis with statistical or graphical software. The methylation status of each case was determined by averaging the values of the 11 CpG sites.

To assess the sensitivity of this assay, we conducted a dilution series experiment using different mixtures of methylated DNA and unmethylated genomic DNA (Zymo Research, Irvine, CA, USA). Intra- and inter-assay reproducibility was evaluated by performing the assay on 6 cases in triplicate within the same run and across three different runs.

Linear regression was performed to assess the relationship between the *BRCA1* methylation level and the input DNA amount. This method allows for quantifying the strength and direction of the linear association between the two variables. The coefficient of determination (R^2^) was calculated to evaluate the proportion of variance in the methylation levels explained by the DNA input amount. A higher R^2^ value indicates a stronger correlation, while a lower value suggests a weaker association. The statistical significance of the regression was also considered to assess the reliability of the observed relationship.

This assay was approved by the New York State Department of Health Clinical Laboratory Evaluation Program (CLEP). The approved assays can be found at this link: https://www.wadsworth.org/regulatory/clep/approved-ldt (accessed on 26 December 2024).

## 3. Results

### 3.1. Design and Interpretation of the Pyrosequencing Assay

We first determined which CpG sites were most suitable for this assay, as many CpG sites are present within the *BRCA1* exon1 promoter region. A literature review identified a region of CpG sites (“Region 1”) that had been tested previously [10].We also had previously performed an alternate assay (Sequenom) that identified a second region of CpG sites (“Region 2”) that was immediately downstream of the first region in intron 1 (Figure 1A). From a set of samples that were previously tested for *BRCA1* promoter methylation using other approaches, we observed relatively less variation in “Region 1”, although both regions gave similar results that were concordant with previous findings (Figure 1B). We therefore chose “Region 1” for clinical validation.

### 3.2. Accuracy

**Figure 2:**
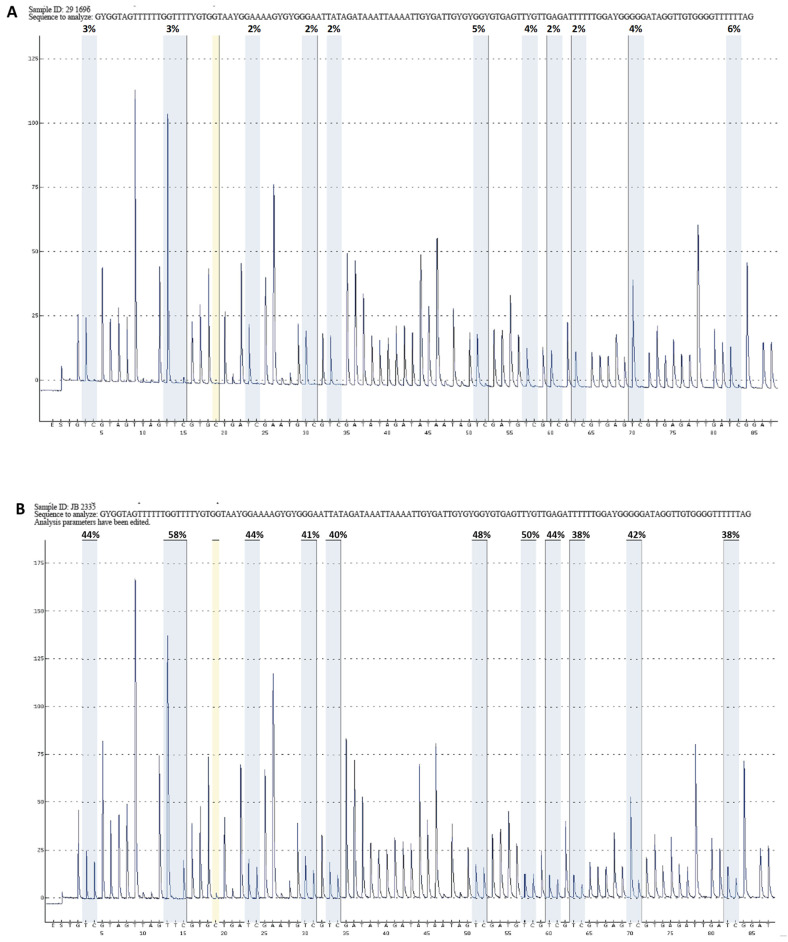
Representative pyrograms of unmethylated ((**A**), sample ID:29-1696 in Table 1) and methylated ((**B**), sample ID: JB-2335 in Table 1) cases. The 11 CpG sites examined by this assay are displayed. The methylation level for each CpG site is calculated and indicated above the sites.

To establish the accuracy of this assay, we selected twenty (20) HGSC cases that were analyzed for methylation events using the Illumina methylation array as part of TCGA project [9] or using a *BRCA1* methylation analysis by a real-time PCR assay as part of this study. Some cases were analyzed using both methods. Ten cases were previously tested and shown to be *BRCA1*-methylated, and ten cases were unmethylated. The methylation results obtained from the pyrosequencing assay were concordant with the previous findings (Table 1). We did not have any failures, indicating that the smaller-sized PCR products could be well amplified from fragmented FFPE DNA. All negative cases exhibited an average methylation level of less than 10% across all 11 CpG sites, although CpG sites #2, #6, and #11 gave slightly higher levels of methylation. For positive results, we observed significantly less variation of methylation levels among these 11 CpG sites (Figure 2). These findings indicate that the pyrosequencing assay provides consistent calls for the methylation status at a 10% threshold when considering the average of all eleven 11 CpG sites. Using these criteria, all 20 cases yielded concordant results with previous data. In addition, the commercially available negative control sample was measured at 5.1% and 7.0% in two separate runs (Table 2A). We therefore used 10% as the cutoff for the detection of methylation.

### 3.3. Analytical Sensitivity

**Figure 3:**
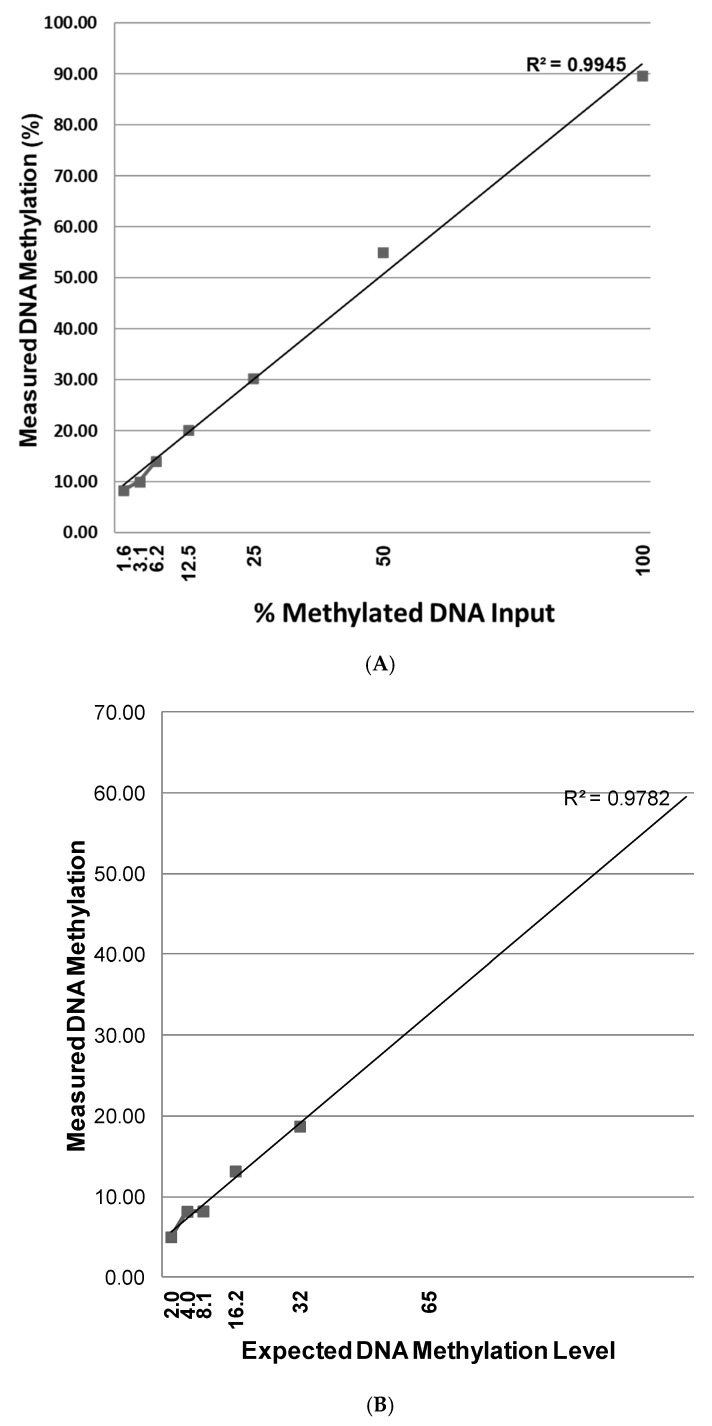
(**A**) Calibration curve for the 11 CpG sites within the core *BRCA1* promoter region using seven mixtures (by DNA amount) of methylated and unmethylated commercial DNA (100%, 50%, 25%, 12.5%, 6.25%, 3.125%, and 1.56%). The curve represents two independent experiments. The readouts are summarized in Table 2A. (**B**) Calibration curve for the 11 CpG sites within the core *BRCA1* promoter region using six mixtures (by DNA amount) of methylated patient DNA samples and unmethylated patient DNA samples (100%, 50%, 25%, 12.5%, 6.25%, and 3.125%). The curve represents two independent experiments. The readouts are summarized in Table 2B.

Results of the sensitivity were summarized in Table 2A,B, as well as in Figure 3A,B. We used the commercially available methylated DNA (Millipore positive control) and one clinical FFPE sample for this analysis. The commercially available methylated DNA was serially diluted with unmethylated DNA to yield varying levels of methylated DNA in each mixture (100%, 50%, 25%, 12.5%, 6.25%, 3.125%, and 1.56%). The mixed samples were prepared prior to the bisulfite treatment and processed as independent samples throughout the entire procedure. Overall, there was a high degree of correlation (*r* > 0.9945) (Figure 3A). The methylation levels were slightly higher than the predicted ratio of methylated/unmethylated DNA at 50%. This higher-than-expected ratio could be due to preferential PCR amplification of the methylated allele and/or the nature of the dilution/mixing experiment. Despite these quantitative differences between predicted and observed results, an overall high degree of correlation was observed (R^2^ = 0.9945). We also performed a dilution series experiment using six mixtures of methylated patient DNA samples and unmethylated matched DNA samples (100%, 50%, 25%, 12.5%, 6.25%, and 3.125%). This was performed to verify that patient DNA samples, extracted from FFPE tissue, could yield a sensitivity similar to the commercially methylated DNA. There was a high degree of correlation (R^2^ = 0.9782) (Figure 3B). These data suggest that the pyrosequencing assay provides concordant calls for the methylation status when a 10% methylation is applied.

### 3.4. Intra- and Inter-Run Reproducibility

Intra-Run reproducibility was performed on six (6) patient samples that were tested in triplicate within the same run. These are technical replicates, as each sample was repeated three times under the same experimental condition. As we included tumor samples from six difference individuals, these data also represent biological replicates. Inter-run reproducibility was performed on six (6) patient samples that were tested in three different runs. Thus, we had three technical replicates and six biological replicates. Both experiments used a mix of samples that were either highly methylated or unmethylated. A hundred percent concordance was reached in both the intra- and inter-run reproducibility tests (Table 3A,B).

## 4. Discussion

PARPis have revolutionized the treatment of OC, and biomarker testing is essential in newly diagnosed patients to identify those who are most likely to benefit from PARPi maintenance therapy and to inform treatment decisions [2,11]. Although selection of patients for PARPis was initially focused on patients with germline and/or somatic *BRCA* mutations, recent studies have shown that *BRCA1*-methylated OC shares molecular similarities with *BRCA*-mutated OC with *BRCA1* silencing. These data suggest that OC with *BRCA1* methylation might be sensitive to PARPi treatment [3,5].

Data for the efficacy of PARPis in the context of *BRCA1*-methylated tumors have been reported in recent years. Olaparib showed limited benefit in a small subset analysis of relapsed tumors with *BRCA1* promoter methylation [12]. Recent exploratory biomarker analyses of ARIEL2 trial samples have revealed that high levels of *BRCA1* methylation serve as a robust predictor of response to rucaparib. This benefit was limited to tumors with homozygous *BRCA1* methylation using a zygosity-determining approach that is not common for stratifying patients for treatment selection [13]. Thus, the availability of the *BRCA1* promoter methylation assay will aid in identifying patients whose tumors lack *BRCA* mutations but have both *BRCA1* alleles methylated. These patients could potentially benefit from treatment with PARP inhibitors like rucaparib.

Additionally, the *BRCA1* promoter methylation may provide a more precise biomarker for identifying homologous recombination deficiency compared to genomic scars. While genomic scarring remains persistent and lacks real-time prediction of sensitivity after prior lines of treatment, methylation status represents a dynamic mechanism that can be evaluated through quantitative methylation assessments [4,14].

PARPis have also been used to treat other cancers, notably breast, prostate, and pancreatic cancers [15,16,17]. Many of these studies have focused on tumors with *BRCA1/2* inactivating mutations and have also shown a PFS benefit. These studies could be expanded to identify patients with *BRCA1* hypermethylation, even though they are classified as *BRCA1/2* wildtype. A recent study evaluated *BRCA1* promoter methylation by quantitative methylation-specific PCR and demonstrated that triple-negative breast cancer (TNBC) patients with a *BRCA1* or *BRCA2* mutation, or high *BRCA1* promoter methylation, showed better 6-month PFS compared with the other patients (*p* = 0.009). Quantitative methylation analysis suggested that the addition of homozygous *BRCA1* promoter methylation to mutations may more accurately identify TNBC patients who would benefit from olaparib/eribulin combination therapy [6]. *BRCA1* promoter methylation measured by MSP was positively associated with the advanced stage of disease and Gleason scores in prostate cancer. *BRCA1* gene expression was significantly downregulated in methylated tumor samples as compared to non-methylated tumors and normal tissues, which suggested that promoter hypermethylation of the *BRCA1* gene could serve as a viable biomarker for prostate cancer [18]. *BRCA1* promoter methylation was not found in two studies, suggesting it is a rare event in pancreatic cancer [19,20].

We designed a *BRCA1* methylation assay using pyrosequencing to aid in stratifying patients whose tumors may be treated with PARPis. This assay is quantitative, reproducible, accurate, fast, and easy to use. The novel aspects of this work are as follows: (1) we conducted a comparison of two regions in the *BRCA1* promoter that could potentially be used for this assay and demonstrated that “Region 1” is more effective in distinguishing *BRCA1* methylation negative cases from positive ones; (2) we analyzed 11 CpG sites using a single PCR reaction, whereas Sahnane et al. reported two different pyrosequencing assays—one analyzing eight CpG sites with two PCR amplicons and two pyrosequencing primers, and another analyzing fourteen CpG sites with two PCR amplicons and three pyrosequencing primers. As a result, our assay is more efficient, as it allows for the analysis of 11 representative CpG sites using just one PCR reaction and one pyrosequencing primer [21]. Pyrosequencing is advantageous over methylation-specific PCR (MSP), as it provides quantitative and more accurate analysis of methylation, especially when analyzing multiple loci is needed. Compared to NGS-based approaches, pyrosequencing is more cost-effective, faster, and less technically demanding. Pyrosequencing is an excellent choice for studies focusing on specific loci or regions and requiring faster results with moderate costs. Although pyrosequencing is a popular method for methylation analysis, it has several disadvantages: (1) limited throughput: pyrosequencing can be time-consuming and may not be suitable for analyzing a large number of samples or a wide range of regions in a single experiment, especially when compared to high-throughput methods like next-generation sequencing (NGS); (2) limited to short regions: pyrosequencing is generally better suited for analyzing small-to-medium-sized DNA regions. It may not be practical for analyzing long genomic regions or large-scale epigenetic profiling; (3) technical challenges: the technique requires precise optimization of conditions, such as primer design and PCR amplification, and issues with PCR biases or sequencing errors can sometimes affect the accuracy of methylation measurements.

Although our validation was conducted with ovarian tumors, this method has applicability to other cancer types. *BRCA1* methylation results could be integrated into routine clinical practice to help select patients for treatment or clinical trials using PARPis, potentially improving PFS for some patients and serving as maintenance therapy for others.

## 5. Conclusions

The PyroMark Q24 pyrosequencer assay had 100% concordance for all ovarian cancer cases included in this validation. This assay has been approved by the New York State Department of Health as a laboratory-specific assay for clinical use.

## Figures and Tables

**Table 1:** Results of *BRCA1* methylation validation and accuracy studies.

	Pos. 1	Pos. 2	Pos. 3	Pos. 4	Pos. 5	Pos. 6	Pos. 7	Pos. 8	Pos. 9	Pos. 10	Pos. 11				
ID	Meth. (%)	Meth. (%)	Meth. (%)	Meth. (%)	Meth. (%)	Meth. (%)	Meth. (%)	Meth. (%)	Meth. (%)	Meth. (%)	Meth. (%)	Mean	Pyro Result	TCGA Result	Real-Time PCR Result
29 1694	55.2	71.3	53.6	49.9	54.1	53.1	58.6	53.9	48.5	43.8	43.3	53.2	PRESENT	METH	
29 1707	50.6	65.9	48.3	45.6	49.2	50.3	53.3	47.2	42.4	41.1	39.4	48.5	PRESENT	METH	
29 1711	61.9	71.8	58.7	55.9	58.7	58.5	65.4	61.2	58.8	52.5	53.6	59.7	PRESENT	METH	
JB 2335	44.1	58.0	44.5	40.9	39.5	47.6	50.1	44.4	37.6	42.1	38.5	44.3	PRESENT	METH	Positive
JB 2595	28.0	51.1	36.7	31.3	28.9	34.7	42.7	43.1	30.1	29.6	25.4	34.7	PRESENT	METH	Positive
JB 2670	62.3	85.6	62.4	61.1	67.0	63.8	69.5	65.6	60.2	58.4	56.3	64.7	PRESENT	METH	Positive
JB 1412	36.3	59.6	41.2	40.4	38.9	40.5	47.4	42.2	34.9	38.2	36.0	41.4	PRESENT		Positive
JB 1468	44.1	61.3	41.2	40.8	42.4	42.8	48.1	42.4	35.0	37.5	29.0	42.2	PRESENT		Positive
JB 3081	41.1	57.7	43.8	39.4	45.0	44.5	48.0	42.3	38.1	36.5	35.5	42.9	PRESENT		Positive
JB 3393	32.8	49.3	34.5	32.0	31.9	34.9	37.1	31.8	27.9	22.4	25.1	32.7	PRESENT		Positive
29 1696	2.8	8.8	2.4	2.3	1.8	4.9	3.6	2.2	1.8	3.9	6.2	3.7	Not Present	Not Meth	
29 1710	2.5	9.8	2.5	3.1	2.4	5.8	3.9	2.1	3.1	3.9	6.2	4.1	Not Present	Not Meth	
29 1762	1.8	11.4	2.8	2.1	2.1	6.9	4.1	1.9	1.5	3.0	7.2	4.1	Not Present	Not Meth	
29 1763	3.8	24.6	7.3	6.4	3.7	11.8	8.8	5.2	4.6	8.9	11.5	8.8	Not Present	Not Meth	
29 1776	3.0	13.1	3.4	2.8	2.8	8.0	5.2	3.3	3.9	6.6	9.5	5.6	Not Present	Not Meth	
29 1784	2.2	9.7	3.8	2.9	2.5	6.5	4.6	2.4	2.0	4.0	7.3	4.4	Not Present	Not Meth	
BRME 23	0.7	12.5	2.4	1.9	1.6	7.0	3.1	2.2	1.0	3.6	7.3	3.9	Not Present		Negative
JB 2206	1.8	10.8	1.7	1.5	1.5	4.5	2.9	1.2	1.4	2.9	5.3	3.2	Not Present		Negative
JB 3717	1.5	10.0	1.6	2.0	1.5	4.2	2.5	1.1	1.5	2.9	4.8	3.1	Not Present		Negative
JB 2388	2.1	9.5	3.0	3.0	2.2	5.4	4.2	2.5	1.9	4.5	6.5	4.1	Not Present		Negative

**Table 2:** (**A**): Results of *BRCA1* methylation sensitivity studies using purified control DNA. (**B**): Results of *BRCA1* methyation sensitivity studies using patient DNA samples.

(**A**)															
		**Pos. 1**	**Pos. 2**	**Pos. 3**	**Pos. 4**	**Pos. 5**	**Pos. 6**	**Pos. 7**	**Pos. 8**	**Pos. 9**	**Pos. 10**	**Pos. 11**			
**Dilution**		**Meth.** **(%)**	**Meth.** **(%)**	**Meth.** **(%)**	**Meth.** **(%)**	**Meth.** **(%)**	**Meth.** **(%)**	**Meth.** **(%)**	**Meth.** **(%)**	**Meth.** **(%)**	**Meth.** **(%)**	**Meth.** **(%)**	**Mean**		
Neg.	Run#1	1.4	14.5	3.3	3.2	2.1	7.0	5.1	2.4	2.2	5.5	8.9	5.1		
Neg.	Run#2	5.1	17.8	5.8	4.0	3.4	8.6	6.7	3.0	3.4	7.3	11.4	7.0		
100%	Run#1	95.0	100.0	90.1	79.7	91.4	90.1	95.1	91.3	92.0	79.4	81.2	89.6		
100%	Run#2	94.9	100.0	91.3	83.1	93.7	90.6	93.9	91.3	88.5	86.7	74.4	89.9		
50%	Run#1	55.6	74.2	55.2	49.3	54.2	57.3	62.4	57.7	52.4	49.1	52.1	56.3		
50%	Run#2	55.5	67.1	53.0	49.2	52.2	53.9	59.1	55.0	50.8	47.1	48.8	53.8		
25%	Run#1	29.3	41.9	28.2	26.5	27.5	30.9	34.5	30.3	27.0	25.9	28.7	30.0		
25%	Run#2	30.6	44.3	30.0	28.3	28.5	30.5	34.0	26.6	26.6	27.8	29.1	30.6		
12.50%	Run#1	18.0	32.4	18.8	18.7	17.9	22.5	23.4	20.0	16.7	18.3	21.2	20.7		
12.50%	Run#2	16.1	30.9	17.2	18.8	17.6	21.3	22.1	18.6	14.5	17.1	21.4	19.6		
6.25%	Run#1	13.0	24.1	12.3	12.5	11.2	15.9	16.1	12.2	11.3	12.9	15.9	14.3		
6.25%	Run#2	13.6	23.3	12.5	12.1	10.0	15.5	14.8	11.6	9.9	11.8	16.4	13.8		
3.13%	Run#1	8.2	18.5	8.6	7.8	7.5	12.0	11.1	8.1	6.6	10.1	13.1	10.1		
3.13%	Run#2	7.5	19.8	8.2	7.9	7.4	11.2	10.5	8.2	6.3	8.9	12.6	9.9		
1.56%	Run#1	5.5	20.3	5.7	6.0	4.3	10.3	8.9	5.5	4.5	7.2	12.0	8.2		
1.56%	Run#2	6.0	20.4	7.4	6.2	4.9	10.2	8.4	5.5	4.7	7.6	11.6	8.4		
(**B**)															
		**Pos. 1**	**Pos. 2**	**Pos. 3**	**Pos. 4**	**Pos. 5**	**Pos. 6**	**Pos. 7**	**Pos. 8**	**Pos. 9**	**Pos. 10**	**Pos. 11**			
**Dilution**		**Meth.** **(%)**	**Meth.** **(%)**	**Meth.** **(%)**	**Meth.** **(%)**	**Meth.** **(%)**	**Meth.** **(%)**	**Meth.** **(%)**	**Meth.** **(%)**	**Meth.** **(%)**	**Meth.** **(%)**	**Meth.** **(%)**	**Mean (measured)**	**Expected**	**Pyro** **Result**
100%	Run#1	59.5	82.9	57.4	55.6	62.5	59.3	69.6	61.5	54.0	54.7	56.1	61.2		DETECTED
100%	Run#2	72.0	87.1	70.2	61.0	71.8	71.0	80.5	67.8	65.3	54.2	61.4	69.3		DETECTED
50%	Run#1	18.9	25.6	18.0	17.3	18.0	18.2	21.4	19.5	14.2	16.2	13.2	18.2	32.5	DETECTED
50%	Run#2	19.7	31.2	21.4	19.0	16.8	21.7	21.7	16.7	12.5	13.2	18.5	19.3	32.5	DETECTED
25%	Run#1	13.0	19.1	12.9	13.6	12.3	15.1	18.2	14.0	10.7	11.4	13.7	14.0	16.2	DETECTED
25%	Run#2	9.6	22.9	10.6	11.0	10.1	14.9	14.5	12.3	7.5	9.1	13.4	12.4	16.2	DETECTED
12.50%	Run#1	6.4	14.7	5.4	4.7	4.9	8.8	7.2	5.4	2.2	6.8	9.3	6.9	8.1	NOT DETECTED
12.50%	Run#2	7.7	16.9	8.6	7.5	6.7	11.2	12.1	7.9	5.0	8.9	12.9	9.6	8.1	NOT DETECTED
6.25%	Run#1	8.1	14.9	7.8	8.1	7.0	10.5	7.6	3.8	3.4	5.2	9.0	7.8	4.0	NOT DETECTED
6.25%	Run#2	6.2	16.9	7.3	8.7	4.9	11.6	9.9	5.9	4.4	8.2	10.9	8.6	4.0	NOT DETECTED
3.13%	Run#1	4.1	12.8	3.2	3.8	4.6	5.9	5.6	3.2	2.2	3.3	8.7	5.2	2.0	NOT DETECTED
3.13%	Run#2	3.3	11.4	5.4	3.6	3.5	6.2	4.1	2.6	2.6	4.4	6.0	4.8	2.0	NOT DETECTED

**Table 3:** (**A**): Results of *BRCA1* methylation intra-assay reproducibility studies. (**B**): Results of *BRCA1* methylation inter-assay reproducibility studies.

(**A**)															
			**Pos. 1**	**Pos. 2**	**Pos. 3**	**Pos. 4**	**Pos. 5**	**Pos. 6**	**Pos. 7**	**Pos. 8**	**Pos. 9**	**Pos. 10**	**Pos. 11**		
**Case #**	**ID**		**Meth.** **(%)**	**Meth.** **(%)**	**Meth.** **(%)**	**Meth.** **(%)**	**Meth.** **(%)**	**Meth.** **(%)**	**Meth.** **(%)**	**Meth.** **(%)**	**Meth.** **(%)**	**Meth.** **(%)**	**Meth.** **(%)**	**Mean**	**Result**
21	JB 1412		50.6	84.4	49.9	48.3	49.7	54.4	53.9	51.8	40.8	44.1	45.8	52.2	PRESENT
21	JB 1412		36.3	59.6	41.2	40.4	38.9	40.5	47.4	42.2	34.9	38.2	36.0	41.4	PRESENT
21	JB1412		50.9	76.8	47.5	44.3	46.1	49.1	55.4	49.8	45.6	44.7	45.6	50.5	PRESENT
22	JB 1468		41.9	63.4	39.6	39.4	40.6	41.3	47.4	41.7	35.7	37.4	28.5	41.5	PRESENT
22	JB 1468		42.0	62.0	41.0	38.5	39.5	40.7	46.7	41.7	36.2	36.2	29.4	41.3	PRESENT
22	JB 1468		43.0	60.4	41.6	40.9	42.5	41.4	47.8	42.2	35.6	39.0	29.9	42.2	PRESENT
23	JB 2400		3.0	13.1	3.3	2.5	1.6	6.5	4.3	1.8	2.4	4.2	7.3	4.5	Not Present
23	JB 2400		3.4	12.0	4.2	3.2	2.5	7.6	4.9	2.6	1.7	4.9	6.7	4.9	Not Present
23	JB 2400		2.7	14.0	4.0	3.5	1.7	6.8	4.5	2.3	1.9	4.5	7.2	4.8	Not Present
24	JB 2505		61.1	97.5	66.3	63.1	67.4	68.2	75.0	67.2	62.4	61.9	61.0	68.3	PRESENT
24	JB 2505		65.4	90.3	65.3	60.8	66.3	67.1	72.4	67.7	61.5	60.8	60.0	67.0	PRESENT
24	JB 2505		62.6	89.7	66.0	58.2	63.6	66.3	71.2	64.3	63.6	60.3	59.0	65.9	PRESENT
25	JB 2520		4.0	13.7	5.0	3.6	2.7	7.0	5.5	3.0	2.5	5.9	8.7	5.6	Not Present
25	JB 2520		2.2	14.5	3.6	2.9	2.4	6.9	4.4	3.1	2.8	4.6	9.0	5.1	Not Present
25	JB 2520		2.7	18.0	3.4	2.2	2.2	6.0	7.3	2.9	2.4	5.1	8.6	5.5	Not Present
26	JB 2540		2.7	12.8	4.0	4.2	3.9	7.5	5.9	3.2	2.1	4.7	7.0	5.3	Not Present
26	JB 2540		5.8	12.3	5.1	4.9	3.2	8.5	5.6	2.7	2.3	5.6	9.6	6.0	Not Present
26	JB 2540		3.7	15.0	5.4	5.1	4.6	7.7	5.7	3.5	3.2	5.9	6.5	6.0	Not Present
(**B**)															
			**Pos. 1**	**Pos. 2**	**Pos. 3**	**Pos. 4**	**Pos. 5**	**Pos. 6**	**Pos. 7**	**Pos. 8**	**Pos. 9**	**Pos. 10**	**Pos. 11**		
**Case #**	**ID**	**Run#**	**Meth.** **(%)**	**Meth.** **(%)**	**Meth.** **(%)**	**Meth.** **(%)**	**Meth.** **(%)**	**Meth.** **(%)**	**Meth.** **(%)**	**Meth.** **(%)**	**Meth.** **(%)**	**Meth.** **(%)**	**Meth.** **(%)**	**Mean**	**Result**
16	BRME 23	Run#1	0.7	12.5	2.4	1.9	1.6	7.0	3.1	2.2	1.0	3.6	7.3	3.9	Not Present
16	BRME 23	Run#2	2.7	11.0	2.9	3.1	0.8	8.3	2.0	0.0	1.5	9.6	9.6	4.7	Not Present
16	BRME 23	Run#3	3.4	17.3	3.9	4.5	5.0	9.1	7.3	4.1	3.4	6.9	8.0	6.6	Not Present
17	JB 1468	Run#1	42.0	62.0	41.0	38.5	39.5	40.7	46.7	41.7	36.2	36.2	29.4	41.3	PRESENT
17	JB 1468	Run#2	28.7	56.0	36.9	35.4	36.1	36.7	37.1	38.5	34.2	33.9	27.6	36.5	PRESENT
17	JB 1468	Run#3	34.3	62.1	40.2	38.7	40.3	42.0	46.4	41.3	33.2	36.7	30.5	40.5	PRESENT
13	JB 2335	Run#1	48.3	63.6	46.7	44.6	44.6	41.6	55.5	41.3	31.6	35.5	36.2	44.5	PRESENT
13	JB 2335	Run#2	44.1	58.0	44.5	40.9	39.5	47.6	50.1	44.4	37.6	42.1	38.5	44.3	PRESENT
13	JB 2335	Run#3	38.4	73.0	47.7	41.2	40.8	48.3	52.8	44.5	35.4	34.6	42.0	45.3	PRESENT
18	JB 2400	Run#1	3.4	12.0	4.2	3.2	2.5	7.6	4.9	2.6	1.7	4.9	6.7	4.9	Not Present
18	JB 2400	Run#2	2.5	12.1	3.8	4.1	3.9	6.4	0.0	0.0	0.0	8.0	4.8	4.1	Not Present
18	JB 2400	Run#3	3.3	8.8	3.5	2.9	3.1	6.0	5.0	4.9	3.1	4.2	4.8	4.5	Not Present
19	JB 2540	Run#1	4.9	16.9	7.0	6.9	3.7	8.2	8.5	4.2	4.8	6.6	11.8	7.6	Not Present
19	JB 2540	Run#2	2.3	10.6	2.8	2.8	3.2	5.3	7.4	3.2	2.8	3.7	7.5	4.7	Not Present
19	JB 2540	Run#3	4.3	28.5	8.2	7.6	5.3	12.5	13.0	6.2	3.9	7.3	11.1	9.8	Not Present
20	JB 3393	Run#1	32.8	49.3	34.5	32.0	31.9	34.9	37.1	31.8	27.9	22.4	25.1	32.7	PRESENT
20	JB 3393	Run#2	32.3	44.7	33.0	33.3	33.1	33.0	39.1	33.9	28.9	24.6	22.1	32.5	PRESENT
20	JB 3393	Run#3	23.6	59.9	32.2	31.1	29.6	35.3	37.9	31.6	24.9	24.8	26.4	32.5	PRESENT

## Data Availability

The original contributions presented in this study are included in the article. Further inquiries can be directed to the corresponding author(s).
